# Study of PLSR-BP model for stability assessment of loess slope based on particle swarm optimization

**DOI:** 10.1038/s41598-021-97484-0

**Published:** 2021-09-09

**Authors:** Bin Gong

**Affiliations:** 1 State Key Laboratory of Coastal and Offshore Engineering, Dalian University of Technology, Dalian, 116024 China; 2 Department of Civil and Environmental Engineering, Brunel University London, London, UB8 3PH UK

**Keywords:** Natural hazards, Civil engineering

## Abstract

The assessment of loess slope stability is a highly complex nonlinear problem. There are many factors that influence the stability of loess slopes. Some of them have the characteristic of uncertainty. Meanwhile, the relationship between different factors may be complicated. The existence of multiple correlation will affect the objectivity of stability analysis and prevent the model from making correct judgments. In this paper, the main factors affecting the stability of loess slopes are analyzed by means of the partial least-squares regression (PLSR). After that, two new synthesis variables with better interpretation to the dependent variables are extracted. By this way, the multicollinearity among variables is overcome preferably. Moreover, the BP neural network is further used to determine the nonlinear relationship between the new components and the slope safety factor. Then, a new improved BP model based on the partial least-squares regression, which is initialized by the particle swarm optimization (PSO) algorithm, is developed, i.e., the PLSR-BP model. The network with global convergence capability is simplified and more efficient. The test results of the model show satisfactory precision, which indicates that the model is feasible and effective for stability evaluation of loess slopes.

## Introduction

The slope stability analysis can not only provide basis for economical and reasonable slope design, but also help to make judgments about the stability state and evolution trend of no matter artificial or natural slopes, prevent the potential risks and guide the slope treatment. Due to the economic development and population expansion, the safety and security of the transport networks and residential areas may be threatened by the potential slope instabilities in many countries. Slope instabilities are complex natural hazards that may result in disastrous consequences^1^. Therefore, the slope stability assessment is a critical research area in civil engineering^2^. In order to ensure the safety of economic construction and prevent the potential economic losses and casualties, slope stability analyses are required, and appropriate assessment methods are of practical need. Currently, the expert evaluation, analytical methods and machine learning are three common methods employed for the slope stability analysis^3,4^. The first method is mainly used to analyze the reasons and developing processes of slope deformation according to the expertise and engineering geological survey. The essence is to apply the previous practical experience into the similar slope engineering projects^5^. Based on the experts’ experiences and knowledge, the relative factors which may trigger the slope collapses can be identified and the safety and stability of a slope can be evaluated. However, the major disadvantage of the expert evaluation techniques is the subjectivity and the decisions may contain the bias of researchers^6^. The analytical methods are mainly used to analyze the slope system characteristics by establishing appropriate mathematical models. Based on this approach, the dangerous sliding surface and safety factor can be identified. However, it is actually difficult to determine the calculation parameters accurately, which may lead to misleading results. In fact, this kind of methods are only appropriate for evaluating slope stability in small areas^7^. Recently, based on the intelligent statistical learning theory, machine learning has been introduced into the slope collapse prediction. The machine learning models are generally established on the basis of the artificial intelligence techniques and historical data^8^. Yan and Li^9^ built a prediction model to evaluate the stability of an open pit slope based on the Bayes discriminant analysis (BDA). Samui and Kothari^10^ applied the least squares support vector machine (LSSVM) to explore the mapping function between the input pattern and the safety factor of slopes. Zhao et al.^11^ developed the nonlinear relationship between the slope stability and influence factors using the relevance vector machine (RVM). Wang et al.^12^ constructed a method to evaluate the stability of complex slope systems based on the projection pursuit algorithm. Liu et al.^13^ applied the improved particle swarm optimization (PSO) algorithm to analyze some critical factors affecting saturated rock slope slip in numerical simulation. Himanshu et al.^14^ used the unified particle swarm optimization (UPSO) to assess optimum location of non-circular failure surface in soil slope. And Moayedi et al.^15^ compared the feasibility of the artificial neural network (ANN), adaptive neuro-fuzzy inference system (ANFIS), and hybrid particle swarm optimization (HPSO) for assessing the safety factor of cohesive slopes.

In terms of loess slope stability evaluation, many internal and external factors should be considered. However, some of them show the obvious features of fuzziness, randomness and variability. Simultaneously, there is a complex nonlinear relationship between the evaluation indices and the influencing factors, which cannot be described by simple mathematical formula. Therefore, the stability assessment of loess slopes is a dynamic, nonlinear, uncertain and systematic problem. Besides, the correlation may exist between different parameters of geomaterials. Actually, it is impossible to take the effects of all the influencing factors into account fully and properly. However, a multi-variable system containing some main factors will be affected by the overlapping information inevitably because the multicollinearity among variables will exaggerate certain characteristics of the analysis system, which will definitely affect the objectivity and chock the decision process^16^.

The artificial neural network (ANN) has been successfully used to solve the slope stability problems by many scholars^17–20^ in recent years. Among them, the back propagation (BP) neural network is a kind of widely used neural networks. BP neural network shows good performance in knowledge learning, experience storage, computational efficiency and fault tolerance. It has the ability to extract features and acquire knowledge from the dynamic uncertain multi-factor systems, and also to approximate any complex nonlinear functional relationship. Meanwhile, in consideration of the explicit error back-propagation strategy and strict weight correction procedure from a mathematical point of view, it is reasonable to evaluate the slope stability with the BP neural network. Partial least-squares regression (PLSR) is a new multivariate statistical data-analysis method which can realize the multiple linear regression, canonical correlation analysis and principal component analysis. Through extracting the representative synthesis variables of a given system, PLSR can reduce the dimension of the independent variable system, simplify the network structure and improve the modeling efficiency. Meanwhile, the adverse effects of the variable multicollinearity can be overcome in this way. Moreover, many evolutionary algorithms were proposed for global optimization and employed to improve the performance of other algorithms, such as the dragonfly algorithm^21^, multi verse optimizer^22^, robust optimization^23^ and cooperative meta-heuristic algorithm^24^. As a population-based global optimization technique, the particle swarm optimization (PSO) has arisen extensive attention from the optimization community because of the simple structure, clear parameter meaning, high convergence rate and little manual intervention. Hence, PSO is adopted to solve the local convergence problem of BP neural network using its excellent global optimization capability, which means the satisfactory global optimal solution can be obtained with a relatively high speed. In this study, by taking advantage of the partial least-squares regression, particle swarm optimization and BP neural network, the PLSR-BP model for loess slope stability assessment is established. And the test results of the model show satisfactory precision and performance.

## Partial least-squares regression

The partial least-squares regression (PLSR)^25^, as the second-generation regression analysis method, was developed for the global data treatment. PLSR can be employed to find the hidden structure of the dataset and extract the meaningful information based on the dimensional reduction of data and inverse calibration technology^26^. Through PLSR, several new representative synthesis variables can be extracted from the variable system by removing the redundant information. By this way, the adverse effects of multicollinearity among variables on the accuracy and reliability of modeling can be overcome effectively^27^.

### Extract principal components using PLSR

Hypothetically, there are ***p*** independent variables ***r*** = {***x***_***1***_*,****x***_***2***_*,…,****x***_***p***_} and ***q*** dependent variables ***s*** = {***y***_***1***_*,****y***_***2***_*,…,****y***_***q***_} (where ***p*** and ***q*** are two positive integers). There are ***n*** groups of independent and dependent variable data, respectively (***X***=${[{{\varvec{r}}}_{1},{{\varvec{r}}}_{2},\cdots ,{{\varvec{r}}}_{{\varvec{n}}}]}_{{\varvec{n}}\times {\varvec{p}}}^{{\varvec{T}}}$ and ***Y*** = ${[{{\varvec{s}}}_{1},{{\varvec{s}}}_{2},\cdots ,{{\varvec{s}}}_{{\varvec{n}}}]}_{{\varvec{n}}\times {\varvec{p}}}^{{\varvec{T}}}$). ***n*** is the number of the selected samples. ***t***_***1***_ and ***u***_***1***_ are extracted from ***X*** and ***Y***, respectively. Namely, ***t***_***1***_ is a linear combination of ***x***_***1***_*, ****x***_***2***_*,…, ****x***_***p***_ and ***u***_***1***_ is a linear combination of ***y***_***1***_*, ****y***_***2***_*, …, ****y***_***q***_. Meanwhile, the following requirements must be met: (1) ***t***_***1***_ and ***u***_***1***_ should carry the variation information of their own data table as much as possible and (2) the correlation degree between ***t***_***1***_ and ***u***_***1***_ should be highest. After extracting the first principal components (***t***_***1***_ and ***u***_***1***_), the partial least-squares regression will be carried out further to get the regression relations of ***X*** and ***t***_***1***_ as well as ***Y*** and ***u***_***1***_. The algorithm will be terminated when the precision of the regression equation is considered to be satisfactory by testing. Otherwise, the residual information of ***X*** and ***Y*** after being explained with ***t***_***1***_ and ***u***_***1***_ will be further used to extract the second components (***t***_***2***_ and ***u***_***2***_). This process repeats until a satisfactory precision is reached.

### Determine principal components using the cross validation (CV)

The principal components of a variable system can be determined by judging whether the prediction ability of the model will be improved significantly when adding the components. The cross-validation method is usually used for the determination of the principal components.

Hypothetically, ***y***_***j***_ represents the sample data and ***t***_***1***_***, t***_***2***_***, …, t***_***A***_ are the principal components extracted by PLSR. ${\widehat{y}}_{hji}$ is the fitted value of ***y***_***j***_ at the ***i***th sample point using the regression model established with ***h*** principal components (***t***_***1***_*, ****t***_***2***_*, …, ****t***_***h***_). These principal components are extracted using all the sample points. ${\widehat{y}}_{hj(-i)}$ is the fitted value of ***y***_***j***_ at the ***i***th sample point using the regression model established with ***h*** principal components (***t’***_***1***_*, ****t’***_***2***_*, …, ****t’***_***h***_). These principal components are extracted using all the sample points except the ***i***th sample point. Moreover, the sum of squared errors ***ss***_***hj***_ of ***y***_***j***_ and the sum of squared prediction errors ***press***_***hj***_ of ***y***_***j***_ are defined as follows^28^:

1 $${ss}_{hj}=\sum_{i=1}^{n}{({y}_{ij}-{\widehat{y}}_{hji})}^{2}$$

2 $${press}_{hj}=\sum_{i=1}^{n}{({y}_{ij}-{\widehat{y}}_{hj(-i)})}^{2}$$

Furthermore, the sum of squared errors ***ss***_***h***_ of ***Y*** which is described by ***h*** principal components extracted from all the sample points and the sum of squared prediction errors ***press***_***h***_ of ***Y*** are defined as follows^28^:

3 $${ss}_{h}=\sum_{j=1}^{q}{ss}_{hj}$$

4 $${press}_{h}=\sum_{j=1}^{q}{press}_{hj}$$

In general, ***press***_***h***_ > ***ss***_***h***_ and ***ss***_***h***_ < ***ss***_***h−1***_. ***ss***_***h−1***_ is the sum of squared errors of ***Y*** which is described by ***h***−1 principal components extracted from all the sample points. Compared with ***ss***_***h−1***_, ***press***_***h***_ reflects not only the role of the principal component ***t***_***h***_, but also the disturbance error of the sample data. Hence, it is always expected that the value of ***press***_***h***_ could be smaller than ***ss***_***h−1***_ to a certain extent (*i.e*., the value of ***press***_***h***_**/*ss***_***h−1***_ is considered to be the smaller the better). Thus, the cross validation of the principal component ***t***_***h***_ can be defined as^28^:

5 $${Q}_{h}^{2}=1-{press}_{h}/{ss}_{h-1}$$

Typically, when ${Q}_{h}^{2}\ge$ 0.0975, the addition of the principal component ***t***_***h***_ will benefit the system; otherwise, there is no need to add the principal component ***t***_***h***_.

### Extraction algorithm of PLSR

The extraction algorithm of PLSR can be summarized as follows:

(1) Complete the standardization process.

The standardization formula is shown as follows^28^:

6 $$\widehat{{z}_{ij}}=({z}_{ij}-\overline{{z }_{j}})/{sd}_{j}$$

where $\widehat{{z}_{ij}}$ is the standardized value, ***z***_***ij***_ is the real value, and $\overline{{z }_{j}}$ and ${sd}_{j}$ are the arithmetic mean and standard deviation of the data in the ***j***th column of the data matrix, respectively.

According to Eq. (6), the standardized data matrices of ***X*** and ***Y*** can be obtained and expressed as ***E***_***0***_ = [***E***_***01***_*,****E***_***02***_*,…,****E***_***0p***_]_***n*×*p***_ and ***F***_***0***_ = [***F***_***01***_*,****F***_***02***_*,…,****F***_***0q***_]_***n*×*q***_. Hypothetically, ***t***_***1***_ and ***u***_***1***_ are the first principal components of ***E***_***0***_ and ***F***_***0***_, respectively.

(2) Extract the principal components.

Calculate the unit eigenvector ***w***_***1***_ corresponding to the largest eigenvalue of the covariance matrix $E_{0}^{T} F_{0} F_{0}^{T} E_{0}$. Note that ***w***_***1***_ is the first axis of ***E***_***0***_ and as a unit eigenvector, *i.e.*, ‖***w***_**1**_‖ = 1. Simultaneously, calculate the unit eigenvector ***c***_***1***_ corresponding to the largest eigenvalue of the covariance matrix $F_{0}^{T} E_{0} E_{0}^{T} F_{0}$. Note that ***c***_***1***_ is the first axis of ***F***_***0***_ and as a unit eigenvector, *i.e.,* ‖***c***_***1***_‖ = 1. After determining the vectors ***w***_**1**_ and ***c***_***1***_, the first principal components can be obtained as ***t***_***1***_ = ***E***_***0***_***w***_***1***_ and ***u***_***1***_ = ***F***_***0***_***c***_***1***_. After that, the two regression equations of ***E***_***0***_ and ***F***_***0***_ about ***t***_***1***_ can be determined, respectively^28^.

7 $${E}_{0}={t}_{1}{a}_{1}^{T}+{E}_{1}$$

8 $${F}_{0}={t}_{1}{b}_{1}^{T}+{F}_{1}$$

where the regression coefficient vectors are as follows^28^:

9 $${a}_{1}={E}_{0}^{T}{t}_{1}/{\| {t}_{1}\| }^{2}$$

10 $${b}_{1}={F}_{0}^{T}{t}_{1}/{\| {t}_{1}\| }^{2}$$

(3) Test the cross validation.

If ${Q}_{h}^{2}\ge$ 0.0975, it means that the next principal components should be extracted. After replacing the residuals matrices ***E***_***0***_ and ***F***_***0***_ with ***E***_***1***_ and ***F***_***1***_, the second principal components ***t***_***2***_ and ***u***_***2***_ can be calculated in the same way. This process will repeat until ${Q}_{h}^{2}<$ 0.0975. If the rank of ***X*** is ***A***, we have the following equations^28^:

11 $${E}_{0}={t}_{1}{a}_{1}^{T}+\dots +{t}_{A}{a}_{A}^{T}+{E}_{A}$$

12 $${F}_{0}={t}_{1}{b}_{1}^{T}+\dots +{t}_{A}{b}_{A}^{T}+{F}_{A}$$

## BP neural network

Artificial neural network^29^ can be seen as a connected parallel architecture consisting of several layers of neurons. For ANN, the knowledge can be gained from the sample sets and be represented as ‘weights’ and ‘thresholds’ in the connections of the neural network. Through the weight and threshold matrices, the influence of the input variables on the output variables can be determined. On the other hand, the appropriate mathematical methods can be chosen to adjust the weights and thresholds to realize specific functions. The BP neural network is used in this study because of its reliability and applicability. According to the randomly initialized weight and threshold matrices, the error between the network output and the target values can be calculated. Then, based on a weight correction procedure, the error is propagated backward and used to update the weight and threshold matrices of the previous layers using the back-propagation algorithm^30^. In this way, the mapping function between the system input variables and output variables can be modelled by the BP neural network step by step.

**Figure 1:**
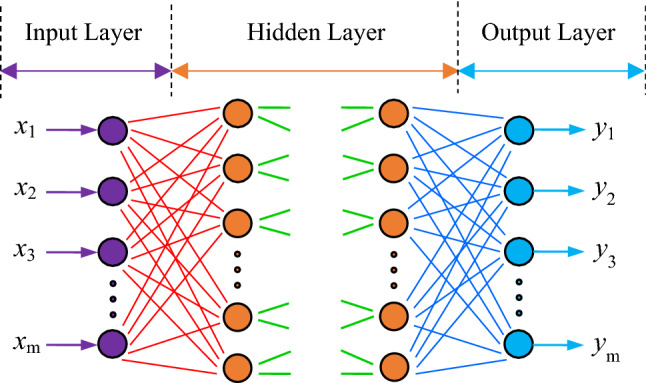
Typical architecture of BP neural network (MS Office 2019; www.microsoft.com/en-us/microsoft-365/get-started-with-office-2019).

The architecture of a standard BP neural network is shown in Fig. 1. Generally, it has one input layer composed of neurons corresponding to the input variables, no less than one hidden layer and one output layer composed of neurons corresponding to the output variables.

In this study, the number of neurons (***m***) in the input layer is the same as the number of physical–mechanical parameters to be considered, and the number of neurons (***n***) in the output layer is the same as the number of evaluation indices. The evaluation index of the slope stability is the safety factor in this paper. Hence, the number ***n*** is 1. The number of neurons (***p***) in a hidden layer can be specified either manually or by an optimization method^30^. The training samples are used to update the weight and threshold matrices by making the summed squared error between the safety factor values and the output of the BP network a minimum using the back propagation algorithm.

**Figure 2:**
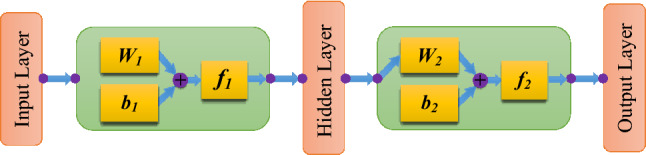
Flow chart of BP neural network (MS Office 2019; www.microsoft.com/en-us/microsoft-365/get-started-with-office-2019).

The computing process of a three-layer BP neural network is shown in Fig. 2. ***W***_***1***_ and ***b***_***1***_ are the weight and threshold matrices between the input and hidden layers, respectively; ***W***_***2***_ and ***b***_***2***_ are the weight and threshold matrices between the hidden and output layers, respectively; ***f***_***1***_ and ***f***_***2***_ are the transfer functions between two adjacent layers. Tan-Sigmoid transfer function (*tansig*), Log-Sigmoid transfer function (*logsig*) and linear transfer function (*purelin*) are the three common transfer functions for multilayer artificial neural networks.

However, the summed squared error between the target values and the output values of the BP network depends on the randomly initialized weight and threshold matrices. The limitations such as slow convergence, local convergence and poor generalization ability hamper the performance of ANN seriously^31^. Therefore, an appropriate optimization algorithm with global optimization capability is necessary for the initial assignment of the weights and thresholds of BP neural network.

## Particle swarm optimization

The particle swarm optimization algorithm (PSO) was proposed by Eberhart and Kennedy^32^. As a new intelligent swarm optimization algorithm, PSO is an important branch of the evolutionary algorithms. The velocity-position model is applied in PSO. The position of each particle represents a candidate solution in the solution space. The quality of the particle solution is measured by the previously defined fitness function.

The position and velocity of Particle ***i*** in the ***n***-dimensional space can be set as ***o***_***i***_ = {***o***_***i1***_*,****o***_***i2***_*,…,****o***_***in***_} and ***v***_***i***_ = {***v***_***i1***_*,****v***_***i2***_*,…,****v***_***in***_}. PSO searches the optimal solution through iterating. Firstly, a group of particles are initialized randomly in the ***n***-dimensional space. The velocity decides the displacement of a particle during one iteration in the solution space. Then, the particle velocity and position are adjusted dynamically according to the individual extremum ***p***_***i***_ = {***p***_***i1***_*,****p***_***i2***_*,…,****p***_***in***_} and global extremum ***g*** = {***g***_***1***_*,****g***_***2***_*,…,****g***_***n***_} using the following formulae^32^:

13 $${v}_{ij}^{k+1}=w{v}_{ij}^{k}+{c}_{1}{r}_{1}\left({p}_{ij}^{k}-{o}_{ij}^{k}\right)+{c}_{2}{r}_{2}\left({g}_{j}^{k}-{o}_{ij}^{k}\right)$$

14 $${o}_{ij}^{k+1}={o}_{ij}^{k}+{v}_{ij}^{k+1}$$

where ***w*** is the inertia weight coefficient, ***c***_***1***_ and ***c***_***2***_ are the learning factors, ***r***_***1***_ and ***r***_***2***_ are the random numbers between (0, 1), ${v}_{ij}^{k}$ and ${o}_{ij}^{k}$ are the ***j***th components of the velocity vector and position vector of Particle ***i*** in the ***k***th epoch, where ***j*** = 1,2,*…,****n*** (***n*** is the dimension of the solution space). The first item of the velocity-updating formula reflects the inheritance of the previous velocity, which makes particles maintain inertial motion; the second item is usually called the cognition term. This item is only related to the particle's own experience and reflects the thinking on behalf of itself; the third item is usually called the social item, which reflects the information sharing and cooperation between particles. By learning from itself and other particles, the particle is targeted to obtain more effective information from its ancestors. This process ensures that the optimal solution can be obtained in a short time^33^.

## The PLSR-BP model for loess slope stability assessment

The stability of loess slopes depends on a lot of internal and external factors involving the soil properties, structure characteristics, groundwater, climate change, weathering effects, seismicity, human activities and so on. The key of assessing the loess slope stability appropriately is to select the influence factors correctly. However, there are no widely accepted theories guiding the selection so far. A common approach is to analyze the practical conditions as well as refer to the experience of the geological experts and engineers. On the basis of analyzing the previous study^34^ and data availability comprehensively, seven parameters are determined as the independent variables affecting the loess slope stability in this study, i.e., density ***γ*** (***x***_***1***_), cohesion ***c*** (***x***_***2***_), internal friction angle ***ϕ*** (***x***_***3***_), slope height ***h*** (***x***_***4***_), slope ratio ***s*** (***x***_***5***_), pore water pressure coefficient ***γ***_***u***_ (***x***_***6***_) and seismic intensity ***q*** (***x***_***7***_). Because the main means analyzing the slope stability quantitatively is to calculate the safety factor, the safety factor ***y***_***1***_ is selected as the dependent variable. The 23 representative loess slopes in Northwest China described in the literature^34^ are selected as the analysis samples, as shown in Table 1.

**Table 1:** Sample data of loess slopes (Gao et al.^34^).

Serial Num	*x*_*1*_	*x*_*2*_	*x*_*3*_	*x*_*4*_	*x*_*5*_	*x*_*6*_	*x*_*7*_	*y*_*1*_
1	17.3	105.8	27.1	81.3	3.2	− 1	0	2.271
2	17.9	72.1	24.1	93.2	3	− 1	7	1.522
3	18.1	63.7	27.5	41.8	2.5	− 1	8	1.657
4	18.7	42.6	22.8	49.7	2.8	− 1	8	1.284
5	18.4	51.1	23.3	34	1.3	0	0	1.445
6	17.3	110	22.8	85.3	2.1	0	7	1.743
7	18.1	63.7	25	81.3	2	0	8	1.384
8	17.5	93.2	27.5	77.4	2.5	0	9	1.611
9	17.7	80.5	24.5	105	2.5	0.25	0	1.519
10	17.7	84.7	24.5	77.4	2	0.25	7	1.379
11	18.3	55.3	24.5	73.4	1.6	0.25	8	1.002
12	18	67.8	24.1	93.2	1.6	0.25	9	0.842
13	17.4	101.6	29.2	65.5	1.6	0.5	0	1.301
14	18.4	51.1	24.5	34	2	0.5	7	1.153
15	18.2	59.5	22.8	49.7	2.3	0.5	8	0.997
16	18.1	63.7	22.4	73.4	2.5	0.5	9	0.77
17	17.5	89.6	25	91.3	1.6	− 1	0	1.28
18	18.7	42.6	22	53.7	1.3	0	8	0.99
19	18.3	70.3	25.7	63.4	2.8	0.25	0	1.59
20	17.3	105.8	28.9	81.3	3.2	− 1	9	1.58
21	18.5	51.1	27.3	34	1.3	0	0	1.73
22	17.7	81.7	24.5	77.4	2	0.25	7	1.36
23	18.2	59.5	22.8	49.8	2.3	0.5	8	0.99

*x*_*1*_, *x*_*2*_, *x*_*3*_, *x*_*4*_, *x*_*5*_, *x*_*6*_, *x*_*7*_ and *y*_*1*_ are the density, cohesion, internal friction angle, slope height, slope ratio, pore water pressure coefficient, seismic intensity and safety factor, respectively.

### Correlation between independent variables

The correlation analysis is carried out on MATLAB platform and the correlation coefficients between different independent variables are shown in Table 2.

**Table 2:** Correlation coefficients between independent variables.

Variable	*x*_*1*_	*x*_*2*_	*x*_*3*_	*x*_*4*_	*x*_*5*_	*x*_*6*_	*x*_*7*_
*x*_*1*_	1	− 0.979**	− 0.492*	− 0.673**	− 0.325	0.211	0.144
*x*_*2*_		1	0.521*	0.627**	0.355	− 0.213	− 0.195
*x*_*3*_			1	0.058	0.200	− 0.291	− 0.314
*x*_*4*_				1	0.288	− 0.163	− 0.025
*x*_*5*_					1	− 0.457*	0.166
*x*_*6*_						1	0.058
*x*_*7*_							1

The sign ‘*’ or ‘**’ indicates that the correlation is significant at 0.05 level or 0.01 level.

It can be seen in Table 2 that the multicollinearity exists among independent variables. Especially, the linear correlations between some variables, such as ***x***_***1***_, ***x***_***2***_, ***x***_***3***_, and ***x***_***4***_ are significant. Thus, it is necessary to overcome the multicollinearity by extracting principal components using the partial least-squares regression.

### Extract principal components by PLSR

The partial least-squares regression is implemented for the system ***y***_***1***_ = ***f***(***x***_***1***_, ***x***_***2***_, ***x***_***3***_, ***x***_***4***_, ***x***_***5***_, ***x***_***6***_, ***x***_***7***_) on MATLAB platform. The cross validation shown in Table 3 demonstrates that the first two principal components ***t***_***1***_ and ***t***_***2***_ are acceptable. The mathematical expression is shown as follows:

15 $$\left[\begin{array}{c}{t}_{1}\\ {t}_{2}\end{array}\right]={\left[\begin{array}{c}\begin{array}{cc}-0.0492& 0.0542\end{array}\\ \begin{array}{cc}0.0567& -0.0260\end{array}\\ \begin{array}{cc}0.0599& 0.0337\end{array}\\ \begin{array}{cc}0.0117& -0.1203\end{array}\\ \begin{array}{cc}0.0438& 0.0266\end{array}\\ \begin{array}{cc}-0.0566& -0.0759\end{array}\\ \begin{array}{cc}-0.0508& -0.1117\end{array}\end{array}\right]}^{T}\left[\begin{array}{c}\widehat{{x}_{1}}\\ \widehat{{x}_{2}}\\ \widehat{{x}_{3}}\\ \widehat{{x}_{4}}\\ \widehat{{x}_{5}}\\ \widehat{\begin{array}{c}{x}_{6}\\ \widehat{{x}_{7}}\end{array}}\end{array}\right]$$

where ${[\widehat{{x}_{1}}\widehat{{,x}_{2},}\widehat{{x}_{3},}\widehat{{x}_{4},}\widehat{{x}_{5,}}\widehat{{x}_{6}},\widehat{{x}_{7}}]}^{T}$ is the standardized matrix of [***x***_***1***_, ***x***_***2***_, ***x***_***3***_, ***x***_***4***_, ***x***_***5***_, ***x***_***6***_, ***x***_***7***_]^T^.

**Table 3:** Cross validation.

Component	*t*_1_	*t*_2_	*t*_3_
${Q}_{h}^{2}$	0.554	0.110	0.015
Threshold	0.0975	0.0975	0.0975

### PLSR-BP model based on the particle swarm optimization algorithm

According to the cross-validation testing, a stability assessment model with good performance can be obtained by only choosing the first two principal components ***t***_***1***_ and ***t***_***2***_. Actually, if the following principal components could not offer more meaningful information for explaining the variable system ***Y***, choosing too many principal components may mislead the understanding about the statistical trend and result in incorrect prediction conclusions.

In this study, the three-layer BP neural network is applied. The two principal components ***t***_***1***_ and ***t***_***2***_ are treated as the input of the neural network, and $\widehat{Y}$ (the standardized value of ***Y***) is treated as the output of the neural network, *i.e.*, there are two neurons at the input layer and one neuron at the output layer. It has been proved that a three-layer BP neural network with ***M*** neurons at the input layer, 2***M*** + 1 neurons at the hidden layer and ***N*** neurons at the hidden layer can express any continuous function accurately^35^. Hence, the structure of the neural network is set to be 2–5–1. Simultaneously, the *logsig* function is applied as the transfer function of the hidden layer and the *purelin* function is applied as the transfer function of the output layer.

**Figure 3:**
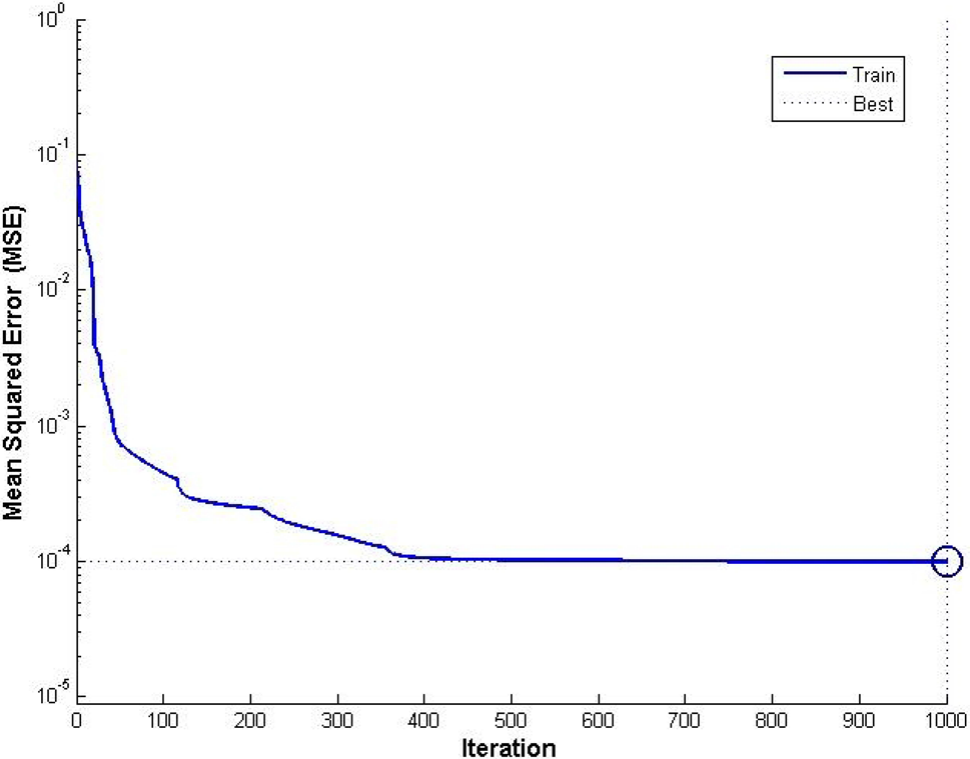
Training process of the neural network.

**Figure 4:**
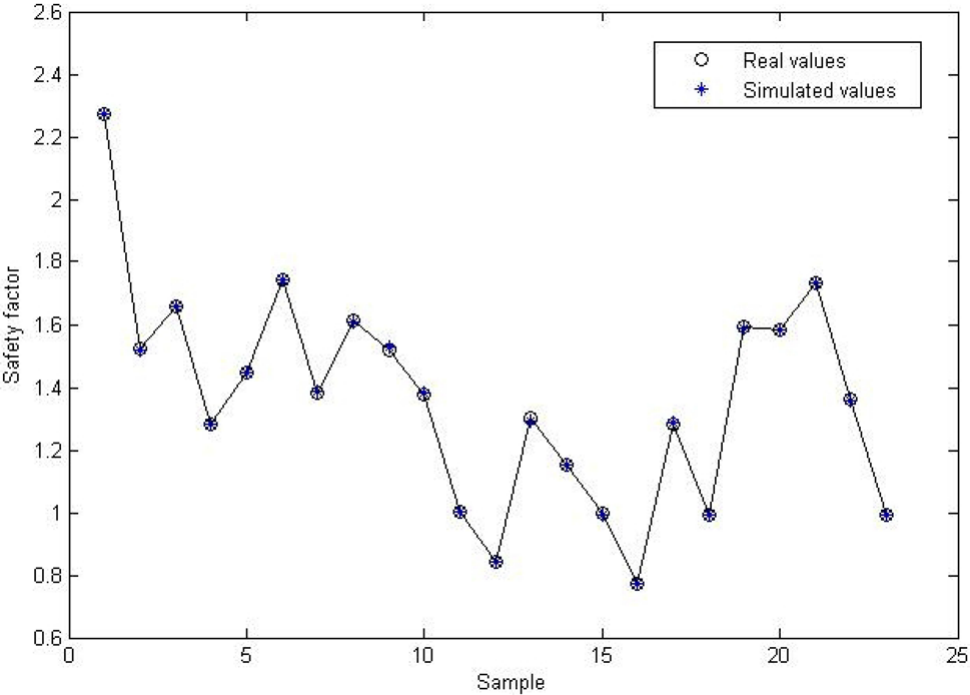
Simulated results of the neural network.

Based on MATLAB platform, the neural network is established by learning knowledge from the 23 groups of sample data listed in Table 1. The initial weights and thresholds are optimized using PSO. Through trial calculation, the parameters of PSO are set as: the total number of particles ***n*** = 23, particle dimension ***d*** = 21, inertia weight coefficient ***w*** decreasing from 1.15 to 0.45 linearly, learning factors ***c***_***1***_ = 2.2 & ***c***_***2***_ = 2.0 and maximum evolution number ***m*** = 200. At the end of evolution, the mean square error (MSE) of the network drops to 0.0787. Then, the neural network is initialized with the optimal particle position and trained with the Levenberg-Marquard algorithm. During this process, the training target is 1 × 10^–5^, maximum iteration number is 1000, learning rate is 0.05, and display interval is 200. At the end of training, the final MSE of the neural network is 9.9667 × 10^–5^. The training process and results are displayed in Figs. 3 and 4. Figure 3 shows that the MSE gradually drops from around 0.8 × 10^–2^ to around 1 × 10^–4^ after 400 iterations and reaches 9.9667 × 10–5 after 1000 iterations, which indicates that the convergence is steady and fast. The total runtime is 30.50893 s on the computer with a i7-10510U CPU and 16 GB RAM. The time complexity of the code is O(***m***), in which ***m*** is the maximum evolution number of PSO. From Fig. 4, we can see that the simulated results of the trained BP neural network almost coincide with the real values, which indicates that the established analysis model can precisely describe the complex nonlinear relationship between the influencing factors and the safety factor and successfully capture the main features of the loess slope stability evaluation system.

Then, four new loess slope samples are used to verify the precision of the established neural network model and the related parameter values are listed in Table 4. Meanwhile, Table 5 shows the comparison of the calculated safety factors by the proposed PLSR-BP model, the PSO-BP model without the partial least-squares regression analysis and the traditional BP neural network. From the evaluation results shown in Table 5, it can be seen that the forecasting model has been significantly improved by extracting the new synthesis variables and overcoming the multicollinearity among variables, and the performance of the established PLSR-BP model is obviously superior to the other two models. Moreover, the maximum absolute error of the output safety factor of the PLSR-BP model is less than 0.040 and the relative error never exceeds 5.0%. The high precision further indicates that the established PLSR-BP model based on the partial least-squares regression is feasible and reliable. Simultaneously, from Table 5, we can see that the relative error of Sample 2 predicted by the PLSR-BP model is largest among all the test samples. That is because the real safety factor of this slope is only 0.790 which is a value lower than 95% of the training samples. Namely, such a low safety factor is not common, and the proposed model hasn’t learned much knowledge about low safety factor because of data availability.

**Table 4:** Parameters of four new loess slope samples.

Serial num	Density /(kN/m^3^)	Cohesion /kPa	Internal friction angle /(°)	Slope height/m	Slope ratio	Pore water pressure coefficient	Seismic intensity	Safety factor
1	17.91	71.35	22.17	52	1.0	0.0	0	1.229
2	17.94	70.13	22.16	73.42	2.48	0.5	9	0.790
3	16.5	100.0	29	45	0.7	0.0	0	1.127
4	18.6	93.0	20	13.2	3.0	0.2	0	1.567

**Table 5:** Comparison between network outputs and practical values.

Serial num	Safety factor	BP			PSO-BP			PLSR-BP		
		Calculated value	Absolute error	Relative error/%	Calculated value	Absolute error	Relative error/%	Calculated value	Absolute error	Relative error/%
1	1.229	1.358	0.129	10.50	1.141	0.088	7.16	1.220	0.009	0.73
2	0.790	1.055	0.265	33.54	0.819	0.029	3.67	0.824	0.034	4.30
3	1.127	1.422	0.295	26.18	1.057	0.070	6.21	1.152	0.025	2.22
4	1.567	1.111	0.456	29.10	1.460	0.107	6.83	1.571	0.004	0.26

## Conclusion

The assessment of loess slope stability is a highly complex nonlinear problem. Some of the factors affecting the slope stability exhibit the characteristics of fuzziness, randomness and variability. Meanwhile, there is a complex nonlinear relationship between the influencing factors and the safety factor. In this study, by taking advantage of the artificial neural network and intelligent swarm optimization algorithm, the improved BP model for the stability assessment of loess slopes is developed based on the partial least-squares regression, i.e., the PLSR-BP model.

Although the stability assessment of loess slopes is a dynamic, nonlinear, uncertain and systematic problem, it has been proved that the BP neural network has the ability to approach the complex nonlinear relationship. It is appropriate and effective to evaluate the loess slope safety and stability using the BP neural network. Moreover, this study focuses on the multicollinearity problem. The correlation analysis indicates that the multicollinearity exists in the variable system. The existence of multiple correlation will affect the objectivity of stability analysis and prevent the model from making correct judgments. Therefore, the partial least-squares regression is carried out and two new synthesis variables with better interpretation to the dependent variables are extracted. In this way, the adverse effects of the variable multicollinearity are overcome. Simultaneously, the neurons at the input layer of BP neural network are also reduced to two, which simplifies the network structure and improves the modeling efficiency. Additionally, with the aim of converging to the global optimal solution more quickly, the BP neural network is initialized by PSO because of its global optimization ability. The test results show satisfactory precision, which indicates that the proposed model is feasible and reliable for the stability evaluation of loess slopes.

Combining the advantages of the particle swarm optimization, BP neural network and partial least-squares regression, the proposed assessment model can not only tackle with the variable correlation, local convergence and nonlinearity problems, but also present more extensive applicability. It can be used to determine the stability state and calculate the safety factor of loess slopes. Meanwhile, more influencing factors, such as rainfall density, groundwater level, weathering degree of geomaterials, etc., can be considered in the developed model based on data availability to conduct parameter sensitivity analysis and form a specific model reflecting the actual situation in a certain area. However, for such a specific model, the quality of the training samples may affect the effectiveness of the established model, *i.e.*, the accurate parameter values should be ensured for the samples. Additionally, the developed model cannot give satisfactory results if the parameter values of an evaluated slope exceed the parameter ranges of the training sample. Although the loess slope stability assessment involves many aspects and a few challenges may be encountered, the proposed model has proved to be an effective and efficient approach for engineers in the field of loess slopes, and it shows potential in a variety of slope engineering applications.

## Data Availability

The datasets generated and/or analyzed during the current study are available from the corresponding author upon reasonable request.
